# Transcriptome Profile and Gene Expression During Different Ovarian Maturation Stages of *Macrobrachium rosenbergii* (De Man, 1879)

**DOI:** 10.21315/tlsr2024.35.3.4

**Published:** 2024-10-07

**Authors:** Mohd Pauzi Mardhiyyah, Muhammad Faiz Zakaria, Adnan Amin-Safwan, Mamat Nur-Syahirah, Yeong Yik Sung, Hongyu Ma, Mhd Ikhwanuddin

**Affiliations:** 1 Higher Institution Centre of Excellence (HICoE), Institute of Tropical Aquaculture and Fisheries (AKUATROP), Universiti Malaysia Terengganu, 21030 Kuala Nerus, Terengganu, Malaysia; 2 Faculty of Science and Marine Environment, Universiti Malaysia Terengganu, 21030 Kuala Nerus, Terengganu, Malaysia; 3 Department of Applied Sciences and Agriculture, Tunku Abdul Rahman University of Management and Technology, 85000 Segamat, Johor, Malaysia; 4 International Institute of Aquaculture and Aquatic Sciences (I-AQUAS), Universiti Putra Malaysia, 71050 Port Dickson, Negeri Sembilan, Malaysia; 5 Institute of Marine Biotechnology, Universiti Malaysia Terengganu, 21030 Kuala Nerus, Terengganu, Malaysia; 6 UMT-STU Joint Shellfish Research Laboratory, Shantou University, Shantou, 515063, China; 7 Guangdong Provincial Key Laboratory of Marine Biotechnology, Shantou University, Shantou, 515063, China; 8 Faculty of Fisheries and Marine, Campus C, Airlangga University, Mulyorejo, Surabaya 60115, Indonesia

**Keywords:** *Macrobrachium rosenbergii*, Ovary Maturation, Transcriptome, Reproduction, Genes Expression, *Macrobrachium rosenbergii*, Pematangan Ovari, Transkriptom, Pembiakan, Ekspresi Gen

## Abstract

*Macrobrachium rosenbergii*, or giant river prawn, is the most economically crucial cultured freshwater crustacean. A predominant challenge in developing crustacean aquaculture is reproduction management, particularly ovary maturation, where identifying regulative mechanisms at the molecular level is critical. Ovary is the primary tissue for studying gene and protein expressions involved in crustacean growth and reproduction. Despite significant interest in *M. rosenbergii*, its gene discovery has been at a relatively small scale compared to other genera. In this study, comprehensive transcriptomic sequencing data for different maturation stages of the ovary of *M. rosenbergii* were observed. The 20 female *M. rosenbergii* samples evaluated were categorised into four maturation stages, 1 to 4. A total of 817,793,14, 841,670,70, 914,248,78 and 878,085,88 raw reads were obtained from stages 1, 2, 3 and 4, respectively. The assembled unique sequences (unigenes) post-clustering (*n* = 98013) was 131,093,546 bp with an average size of 1,338 bp. The BLASTX unigene search against National Centre for Biotechnology Information (NCBI), non-redundant (NR), nucleotide sequence (NT), Kyoto Encyclopaedia of Genes and Genomes Orthology (KO), Swiss-Prot, Protein Family (PFAM), Gene Ontology (GO), and euKaryotic Orthologous Groups (KOG) databases yielded 27,680 (28.24%), 7,449 (7.59%), 13,026 (13.29%), 22,606 (23.06%), 29,907 (30.51%), 30,025 (30.63%) and 14,368 (14.65%) significant matches, respectively, totalling to 37,338 annotated unigenes (38.09%). The differentially expressed genes (DEG) analysis conducted in this study led to identifying cyclin B, insulin receptor (IR), oestrogen sulfotransferase (ESULT) and vitellogenin (Vg), which are critical in ovarian maturation. Nevertheless, some *M. rosenbergii* ovarian maturation-related genes, such as small ubiquitin-like modifier (SUMO)-activating enzyme subunit 1, E3 ubiquitin-protein ligase RNF25, and neuroparsin, were first identified in this study. The data obtained in the present study could considerably contribute to understanding the gene expression and genome structure in *M. rosenbergii* ovaries throughout its developmental stage.

## Abstract (fr)

**Abstrak:**

*Macrobrachium rosenbergii*, atau udang galah, adalah krustasea air tawar yang paling penting daripada segi ekonomi dalam bidang penternakan. Cabaran utama dalam membangunkan akuakultur krustasea adalah pengurusan pembiakan, terutamanya pematangan ovari, di mana mengenal pasti mekanisme pengawalan pada tahap molekul adalah penting. Ovari merupakan tisu utama untuk mengkaji ekspresi gen dan protein yang terlibat dalam pertumbuhan dan pembiakan krustasea. Walaupun terdapat minat yang tinggi terhadap *M. rosenbergii*, penemuan gennya masih pada skala yang agak kecil berbanding dengan genus lain. Dalam kajian ini, data jujukan transkriptom yang menyeluruh untuk pelbagai peringkat pematangan ovari *M. rosenbergii* telah diperhatikan. Sebanyak 20 sampel betina *M. rosenbergii* yang dikaji telah dikategorikan ke dalam empat peringkat pematangan, dari 1 hingga 4. Sebanyak 81,779,314, 84,167,070, 91,424,878 dan 87,808,588 bacaan mentah diperoleh masing-masing daripada peringkat 1, 2, 3 dan 4. Jujukan unik (unigenes) yang disusun selepas pengelompokan (*n* = 98013) adalah 131,093,546 bp dengan saiz purata 1,338 bp. Carian unigene BLASTX di pengkalan National Centre for Biotechnology Information (NCBI), non-redundant (NR), nucleotide sequence (NT), Kyoto Encyclopedia of Genes and Genomes Orthology (KO), Swiss-Prot, Protein Family (PFAM), Gene Ontology (GO) dan pangkalan data euKaryotic Orthologous Groups (KOG) menunjukkan 27,680 (28.24%), 7,449 (7.59%), 13,026 (13.29%), 22,606 (23.06%), 29,907 (30.51%), 30,025 (30.63%) dan 14,368 (14.65%) padanan yang signifikan, masing-masing dengan jumlah keseluruhan 37,338 unigene yang dianotasi (38.09%). Analisis gen yang diekspresikan secara berbeza (DEG) yang dijalankan dalam kajian ini telah mengenal pasti cyclin B, insulin receptor (IR), oestrogen sulfotransferase (ESULT) dan vitellogenin (Vg), yang penting dalam pematangan ovari. Namun begitu, beberapa gen yang berkaitan dengan pematangan ovari *M. rosenbergii*, seperti subunit 1 enzim pengaktif small ubiquitin-like modifier (SUMO), E3 ubiquitin-protein ligase RNF25 dan neuroparsin, telah dikenal pasti buat kali pertama dalam kajian ini. Data yang diperoleh dalam kajian ini boleh menyumbang dengan ketara kepada pemahaman mengenai ekspresi gen dan struktur genom dalam ovari *M. rosenbergii* sepanjang peringkat pembangunannya.

**Highlights**
- Identification of cyclin B, insulin receptor (IR), oestrogen sulfotransferase (ESULT) and vitellogenin (Vg), which are critical in ovarian maturation.
- Comparison between ovary stages 3 and 1 recorded the most total differentially expressed genes (DEG).
- Small ubiquitin-like modifier (SUMO)-activating enzyme subunit 1, E3 ubiquitin-protein ligase RNF25 and neuroparsin were identified for the first time.

## INTRODUCTION

The Malaysian giant river prawn, *Macrobrachium rosenbergii*, is the largest Palaemonid shrimp (De Grave *et al*. 2008; Arockiaraj M *et al*. 2011). The species is among the most diverse freshwater Crustacea genera (De Grave *et al*. 2008; Arockiaraj M *et al*. 2011). Although adult giant river prawns live in freshwater, their larvae require brackish water to develop and survive (Arockiaraj J *et al*. 2011; Arockiaraj M *et al*. 2011). The wide range of salinity in the life cycle of *M. rosenbergii* has led to mass-rearing technique development for commercial production, which allowed culturing in and out of its native range and for aquaculture (Arockiaraj *et al*. 2012). The importance of the prawn species in aquaculture has also initiated research addressing various aspects that directly or indirectly affect its commercial production, including reproduction, fisheries, nutrition, bacterial and viral diseases, and environmental stress responses (Mohd-Shamsudin *et al*. 2013).

Regulating reproduction, specifically ovary maturation, presents a significant issue in crustacean aquaculture development (Martins *et al*. 2007). The ovary produces female gametes through oogenesis and secretes progesterone, directly related to sexual maturation and reproduction. Although information on *M. rosenbergii* ovaries and germ cell morphology, ultrastructure, and histological changes during ovarian development is available (Soonklang *et al*. 2012), regulatory mechanisms and gene expression in ovaries during ovarian maturation remain poorly understood. Ovaries are also the primary tissues for studying genes and protein expressions involved in crustacean growth and reproduction (Suwansa-Ard *et al*. 2015). Despite the remarkable interest in *M. rosenbergii*, its gene discovery has been conducted at a relatively small scale compared to other river prawns of similar genus (Mohd-Shamsudin *et al*. 2013).

Next-generation sequencing (NGS) technologies have revolutionised genomic research. The technique offers considerable sequence data amount delivery capabilities of deeper and wider information in less time and at significantly lower costs than its conventional Sanger counterpart (Mohd-Shamsudin *et al*. 2013). Consequently, NGS has been employed to sequence and characterise various organism organ and cell line transcriptomes (Mohd-Shamsudin *et al*. 2013). RNA sequence (RNA-seq) technology offers qualitative and quantitative gene expression data at superior sensitivity and accuracy than precedent transcriptomic methods, such as expressed sequence tag (EST) sequencing, serial analysis of gene expression (SAGE), massively parallel signature sequencing (MPSS) and microarrays. Furthermore, RNA-seq could reveal actively expressed genes in specific tissues and species of interest. The approach also permits potential molecular marker discoveries, which is particularly useful in non-model organisms without a complete available genome (Mohd-Shamsudin *et al*. 2013).

Transcriptomic analysis has been utilised to establish growth-related genes in *M. rosenbergii* muscle, ovary and testis (Jung *et al*. 2011) and procure differential gene expression profiles in its hepatopancreas, gills and muscle (Mohd-Shamsudin *et al*. 2013). The approach was also employed to analyse the hepatopancreas in response to *Vibrio parahaemolyticus* (Rao *et al*. 2015) and *Vibrio harveyi* (Baliarsingh *et al*. 2021) infections, white spot syndrome virus and poly challenges (Ding *et al*. 2018), and post-larvae responses to nodavirus infection (Pasookhush *et al*. 2019) of *M. rosenbergii*. Jiang *et al*. (2019) reported transcriptomic evaluations on eyestalks of the giant river prawn, revealing ovarian maturation-related genes, while Liu *et al*. (2021) assessed the gills and hepatopancreas of the prawn post-exposure to cadmium (heavy metal). Transcriptomic analysis has also been performed on *M. rosenbergii* gonadal tissues to identify precocious puberty and slow growth (Ying *et al*. 2022).

NGS data on the developmental stages of *M. rosenbergii* ovary is unavailable. Consequently, the present study provided comprehensive transcriptome data derived from varying maturation stages of *M. rosenbergii* ovary tissues by utilising Illumina HiSeq. The primary aim of the current study was to identify candidate genes associated with reproduction and ovarian development in *M. rosenbergii*. This study also established the differential gene expressions from ovarian development phases to better understand their functions.

## MATERIALS AND METHODS

### Sample Preparation and RNA Extraction

**Figure 1:**
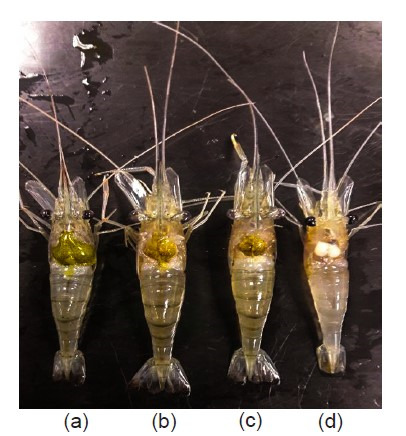
The external morphological view of stages (a) 4, (b) 3, (c) 2 and (d) 1 of *M. rosenbergii* ovarian maturity.

Twenty adult *M. rosenbergii* females weighing between 20 g and 30 g were obtained from Sungai Manir, Kuala Terengganu. The prawns were transported to the hatchery in the Institute of Tropical Aquaculture and Fisheries, Universiti Malaysia Terengganu, Terengganu, Malaysia, before being disinfected and grown briefly in filtered fresh water and sacrificed. Subsequently, the prawn specimens were categorised into four groups according to their ovary maturation phase, divided into stages 1 to 4 (Fig. 1). The classification was based on the external morphology, colour, and gonad-somatic index (GSI) of the reproductive organ. All ovaries were removed and snap-frozen in liquid nitrogen. Five samples were obtained from each maturation stage and stored at −80°C before further analysis.

This study extracted total RNA with innuPREP RNA Mini Kit (Analytik Jena, Germany). The integrity and purity of the total RNA was evaluated utilising the RNA Nano 6000 Assay Kit of the Agilent Bioanalyzer 2100 system (Agilent Technologies, California, US) and the NanoPhotometer spectrophotometer (IMPLEN, California, US). The concentration of the RNA was determined with Qubit® RNA Assay Kit in Qubit® 2.0 Fluorometer (Life Technologies, California, US). The five RNA samples from each stage were pooled in equal amounts, yielding four pooled RNA samples from each maturation phase. The pooled RNA specimens were then employed to prepare four separate RNA-seq transcriptome libraries.

### Transcriptome Library Preparation and Illumina Sequencing

A total of 1.5 μg of RNA from each sample was utilised as input material for specimen preparations before generating sequencing libraries with NEBNext® Ultra™ RNA Library Prep Kit for Illumina® (Nebraska, US) following the manufacturer’s recommendations. Subsequently, the mRNA obtained was purified from the total RNA utilising poly-T oligo-attached magnetic beads. Fragmentation in the present study was performed with divalent cations under elevated temperature in NEBNext First Strand Synthesis Reaction Buffer (5×).

The first strand cDNA was synthesised with random hexamer primer and M-MuLVReverse Transcriptase (RNase H̄), while the second strand cDNA synthesis employed DNA Polymerase I and RNase H. Oxonuclease or polymerase activities were utilised during remaining overhang conversions into blunt ends. Post-adenylation of the 3′ ends of the DNA fragments in the present study, NEBNext Adaptor with hairpin loop structure were ligated as hybridisation preparation. Subsequently, the library fragments were purified with the AmPure XP system (Beckman Coulter, Beverly, US) to select cDNA fragments between 150 and 200 bp.

A total of 3 μL USER Enzyme (Nebraska, US) was employed with size-selected, adaptor-ligated cDNA at 37°C for 15 min followed by 5 min at 95°C. Polymerase chain reaction (PCR) was performed with Phusion High-Fidelity DNA Polymerase (Thermo Fisher Scientific, Massachusetts, US), Universal PCR primers (Bio-Rad, California, US), and Index (X) Primer (New England Biolabs, Massachusetts, US). The PCR products were purified, and library quality was assessed with the Agilent Bioanalyzer 2100 System. Clustering the index-coded samples was performed on a cBot Cluster Generation System utilising a HiSeq PE Cluster Kit cBot-HS (Illumina) per the manufacturer’s instructions. Subsequently, the library specimens were sequenced on an Illumina Hiseq platform and paired-end reads were generated.

### Pre-processing and *de novo* Assembly

In this study, the raw reads were filtered into high-quality clean reads through in-house Perl scripts to ensure downstream analyses were based on clean, high-quality data. During filtration, reads with adaptor contamination, uncertain nucleotides constituting over 10% of read (N > 10%), and low-quality nucleotide (base quality under 20) with more than 50% read were discarded. RNA-seq adapters sequences used in the filtering process was shown in Table 1.

The clean reads procured were assembled *de novo* into transcripts utilising the Trinity programme (Illumina, California, US) (Grabherr *et al*. 2011). The transcripts were clustered with hierarchical clustering (Corset) to eliminate redundant sequences based on sequence similarities. The longest transcript in each cluster represented the final unique sequence (unigene). All unigenes from *M. rosenbergii* samples evaluated in this study were deposited in GenBank, National Centre for Biotechnology Information (NCBI, US, http://www.ncbi.nlm.nih.gov) under the accession number SRP324893.

### Functional Annotation

All unigene yields in the current study were searched against protein databases, including the non-redundant (NR) protein sequence, nucleotide sequence (NT), Swiss-Prot and Protein Family (PFAM) databases, to identify proteins most similar to the newly generated unigenes. Subsequently, the function annotations of similar proteins were retrieved via BLASTX (Novogene, Singapore) with a typical cut-off E-value < 1e^−5^. Biological processes, molecular functions and cellular components were described with unigene gene ontology (GO) annotations by utilising BLAST2GO software version 2.5 (Novogene, Singapore) (Götz *et al*. 2008) at < 1e^−6^ E-value and also euKaryotic Orthologous Groups (KOG). The sequences were assigned to Kyoto Encyclopedia of Genes and Genomes (KEGG) pathways via the online KEGG Automatic Annotation Server (KAAS) (http://www.genome.jp/kegg/kaas/) post-searches in the KEGG genes database.

### Identification and Validation of Differentially Expressed Genes (DEG)

The present study employed the *de novo* transcriptome filterers by Corset as a reference, while the RNA-seq by Expectation Maximisation (RSEM) mapped the reads back to transcriptome and quantified their expression levels. The gene expression levels were quantified by determining the fragments per kilo-base of the exon model per million mapped reads (FPKM). Gene expression levels between samples were also compared based on the FPKM value, calculated as followed:


$$\text{FPKM}=\frac{\text{Total exon fragment}}{\text{Mapped reads }(\text{million})\times \text{Exon length }(\text{kb})}$$

where total exon fragments denote the number of reads aligned to a specific unigene, mapped reads represent the total number of reads aligned to all unigenes, and exon length (kb) is the length of the unigene.

Before conducting the differential gene expression analysis, the read counts factor for each sequenced library was adjusted with one scaling normalised factor utilising an edgeR programme package. On the other hand, differential expression evaluations of two samples were performed with the DEGseq (2010) R package (Novogene, Singapore). The *p*-value in this study was adjusted with *q*-value (Storey 2003) at < 0.005, while |log2 (foldchange)| > 1 was set as the threshold for significantly varied expression. The total RNA from all maturity stages was extracted and purified utilising the innuPREP Mini Kit following the protocol outlined by the manufacturer (Analytic Jena, Germany). The concentration and purity of the total RNA were also spectrophotometrically measured at 260/280 nm. An A260/A280 absorbance ratio over 1.8 indicated remarkable RNA purity.

A 1.2% agarose gel electrophoresis was employed to determine the integrity of the RNA assessed in the present study. The total RNA at a 100 ng/μL concentration was reverse transcribed with a High-Capacity cDNA Reverse Transcription Kit (Applied Biosystems, US) as per the guideline recommended by the manufacturer. First, 2 μL of 10RT buffer, 0.8 μL of dNTP, 2 μL of random primer, 4.2 μL of nuclease H_2_O and 1 μL of MultiScribe®Reverse Transcriptase were prepared. The reaction conditions were 30°C for 10 min, 42°C for 20 min, 99°C for 5 min and 4°C on a Thermal Cycler (Eppendorf, UK). The present study determined the success of the PCR amplification with a 2% agarose gel electrophoresis. The PCR products were employed as the cDNA template in real-time quantitative PCR (RT-qPCR) to validate differentially expressed genes (DEG) obtained from transcriptome data.

The relative vitellogenin (Vg) expression was also established through quantitative PCR (qPCR). All reactions were conducted in triplicates for each sample. The SYBR Green I qPCR assay conducted in this study utilised a 96-CFX (Bio-Rad, US). The amplifications were performed in an eight-strip 0.2 μL tube in a 25 μL reaction volume consisting of 12.5 μL of 2× SYBR Green Master Mix (Bioline, UK), 1 μL of each forward and reverse Vg-primer (8 μM), 1 μL of cDNA template, and 9.5 μL of nuclease-free water. The 18S rRNA (Table 2) was amplified as a housekeeping gene according to the procedures described by Lafontaine *et al*. (2016). The thermal profile for the SYBR Green qPCR was 95°C for 2 min, followed by 40 cycles at 95°C for 5 s and 62°C for 20 s. The target gene transcript (Vg) was normalised with a housekeeping gene transcript (18 S rRNA) according to the 2−ΔΔCT method (Livak & Schmittgen 2001).

## RESULTS

### Sequencing, Assembly and Clustering Output

**Figure 2:**
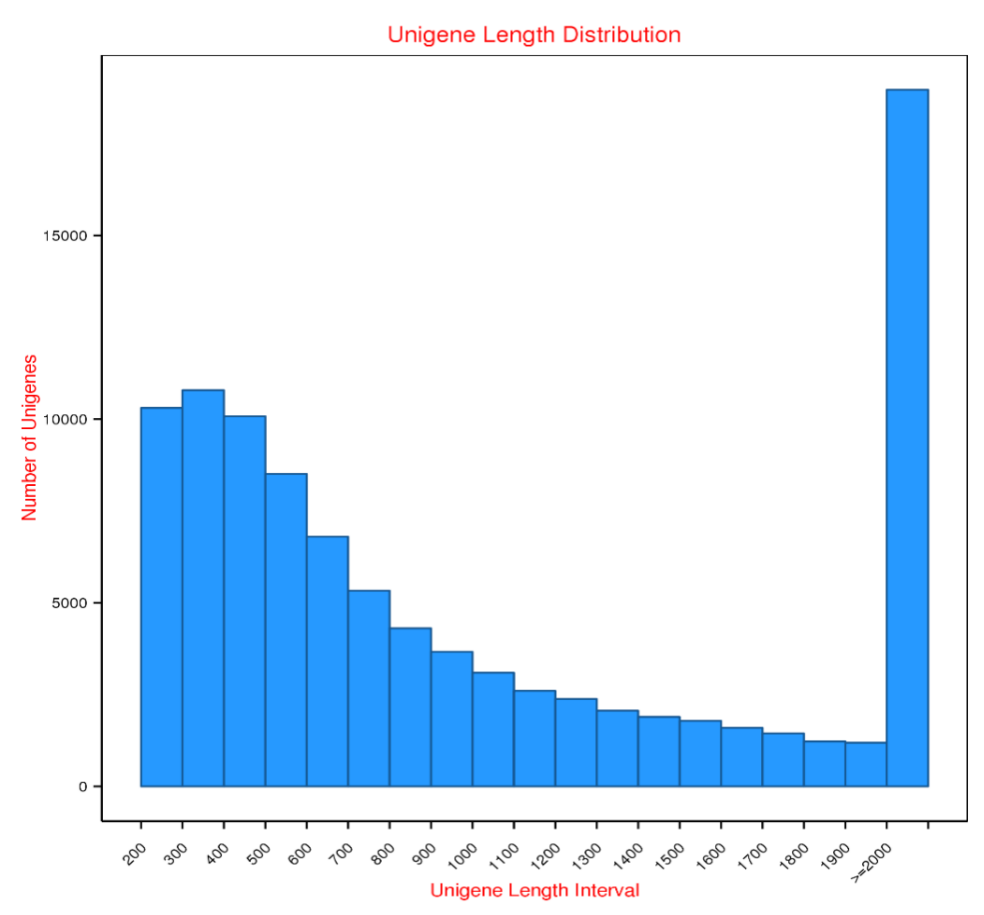
The size distribution of 98,013 unigenes. The abundance of unigenes assembled from ovary stages 1 to 4 libraries based on nucleotide length and the minimum and maximum length of unigene were 201 bp and 24,939 bp, respectively.

Four cDNA libraries representing maturation stages 1 to 4 of *M. rosenbergii* were sequenced with Illumina HiSeq 2000 platform. A total of 817,793,14, 841,670,70, 914,248,78 and 878,085,88 raw reads were obtained from stages 1, 2, 3 and 4, respectively. Approximately 95.50%, 95.69%, 97.47% and 96.04% of clean reads for each stage were retrieved after pre-processing (adaptor removal, quality trimming and N removals), discarding low-quality and empty reads. The assembled unigenes post-clustering (n: 98013) was 131,093,546 bp, had an average size of 1,338 bp and N50 of 2,326 bp, and ranged from 201 to 24,939 bp. Tables 3 and 4 summarise the unigene assembly statistics and length distribution overview, illustrated in Fig. 2. The read data is available in the NCBI Short Read Archive (SRA) under the accession number SRP324893.

### Functional Annotation

**Figure 3:**
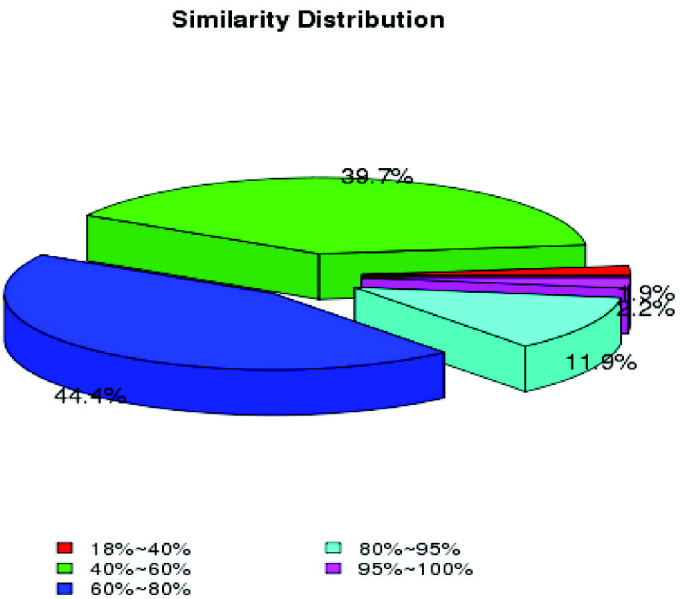
The BLAST hits-based similarity distribution of NR annotation results.

**Figure 4:**
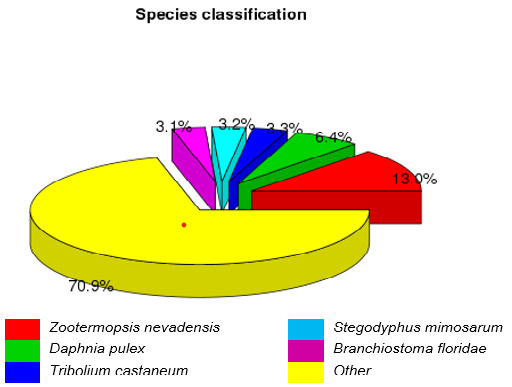
Species distribution of the top five BLAST hits against the NR database.

The unigene BLASTX search against the NCBI NR protein sequence, NCBI nucleotide sequence (NT), KEGG orthology (KO), Swiss-Prot, PFAM, GO and KOG databases returned 27,680 (28.24%), 7,449 (7.59%), 1,026 (13.29%), 22,606 (23.06%), 29,907 (30.51%), 30,025 (30.63%) and 14,368 (14.65%) significant matches, respectively, totalling 37,338 annotated unigenes (38.09%) (Table 5). Fig. 3 shows the blast hit similarity distribution. Approximately 98.2% of the top hit alignments recorded likeness over 40%. The NR database demonstrated that the Nevada termite, *Zootermopsis nevadensis* had the highest matched assembled percentage (13.0%), followed by water flea, *Daphnia pulex* (6.4%), red flour beetle, *Tribolium castaneum* (3.3%), the African social velvet spider, *Stegodyphus mimosarum* (3.2%) and the Florida lancelet, *Branchiostoma floridae* (3.1%) (Fig. 4 and Table 6). The top 20 *M. rosenbergii* annotated transcriptomes based on E-value and hit score are listed in Table 7.

### GO Assignments and KOG Analysis

**Figure 5:**
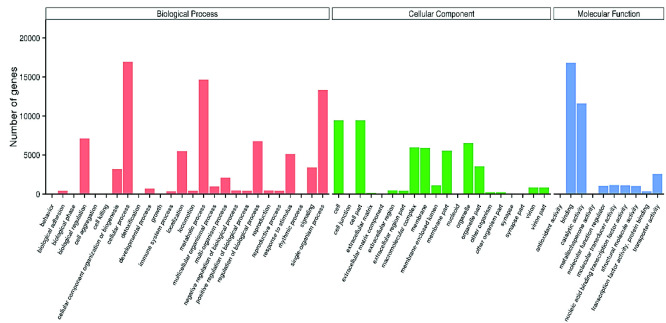
The GO classification of the 30,025 protein annotated unigenes. *Note*: The unigene were classified into GO sub-categories: biological process (red), cellular component (green), and molecular function (blue), and each bar represents the relative abundance of unigenes classified under each sub-category.

**Figure 6:**
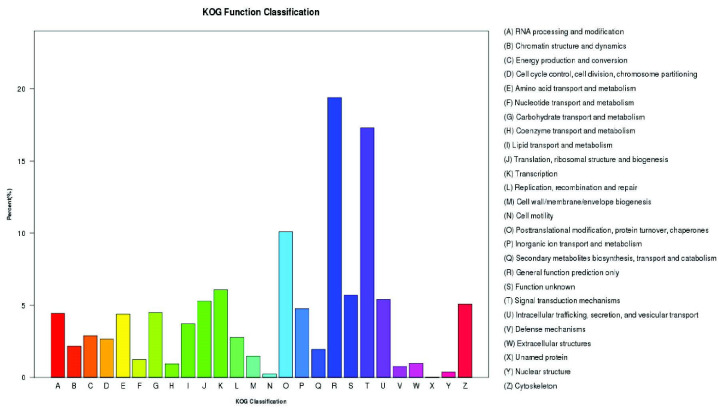
The KOG-based classification of 14,368 known protein annotated unigene histogram. *Note*: Each bar denotes the number of unigenes classified into each of the 26 KOG functional categories.

The GO-based unigene classification resulted in 30,025 (30.63%) unigenes categorised into three domains: biological process (82,862), cellular component (50,606) and molecular function (35,667). Fig. 5 illustrates the distribution of genes for each domain. The top three categories in the biological process domain were “cellular process” (16,925), “metabolic process” (14,669) and “single-organism process” (13,360). In the GO cellular component domain, most of the transcripts were involved in “cell” (9,453), “cell part” (9,453) and “organelle” (6,512), while the molecular function domain documented “binding” (16,819), “catalytic activity” (11,585) and “transporter activity” (2,571) as the top three. Based on Fig. 6, the KOG classification based on the BLAST search against the database resulted in 14,368 unigenes categorised into 26 categories, with the highest number of unigenes grouped under general function prediction (2,786), followed by signal transduction mechanisms (2,485), transcription (871), function unknown (817), intracellular trafficking, secretion, and vesicular transport (775), translation, ribosomal structure and biogenesis (758) and cytoskeleton (730).

### The KEGG Analysis

**Figure 7:**
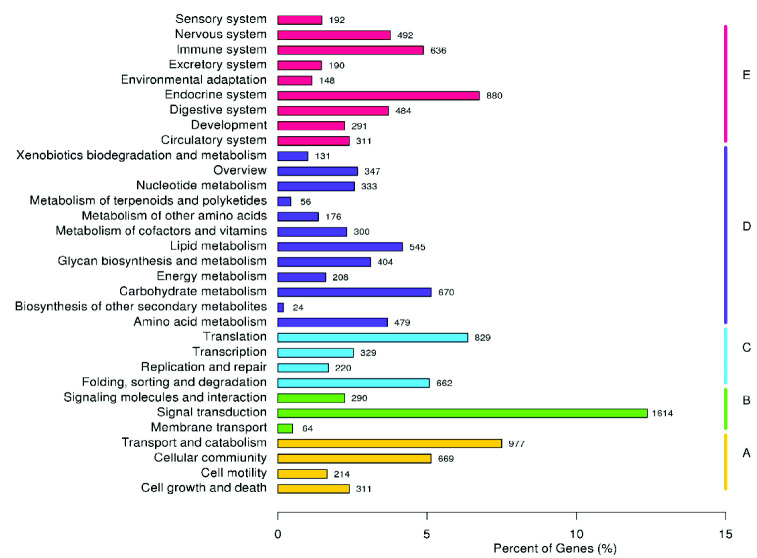
The KEGG biological pathway classification histograms of 13,026 protein annotated unigenes. *Note*: Each bar indicates the number of unigenes systematically categorised into sub-classes of A: cellular processes, B: metabolism, C: genetic information processing, D: environmental information processing, and E: cellular processes.

Systematic categorisation of the unigenes based on the KEGG biological pathway revealed 13,026 unigenes mapped to 230 KEGG pathways. In the first hierarchy level, the transcriptome data encompassed four cellular processes, three environmental information and four genetic information processing, and 12 metabolism and nine organismal systems. The unigene distribution across the second KEGG pathway hierarchy level is illustrated in Fig. 7. The most specific categorical level exhibited 1,614 unigenes mapped to signal transduction, 977 to transport and catabolism, 880 for the endocrine system and 829 to translation.

### Identification of Reproduction and Ovary Development-associated Genes and Validation of Differentially Expressed Gene

**Figure 8:**
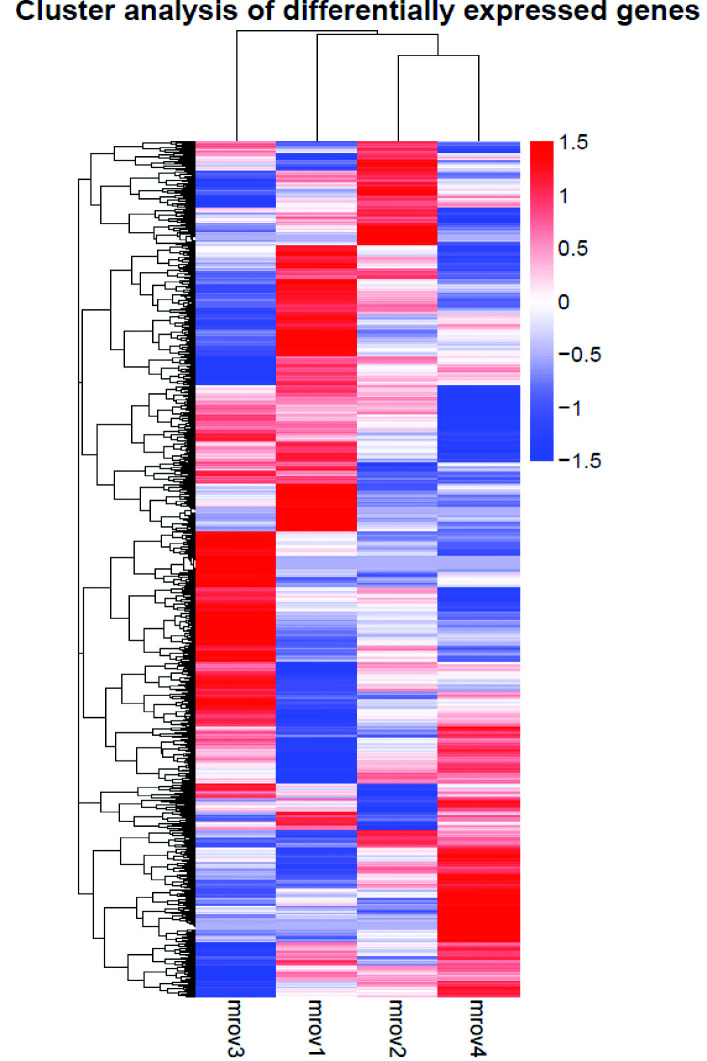
The gene expression value heatmap depicting gene clustering between stages 3 (mrov3, first left panel), 1 (mrov1, second left panel), 2 (mrov2, third panel), and 4 (mrov4, right panel) based on the expression of mRNAs for a set of significant genes. *Notes*: Sample names are represented in columns, and significant genes are denoted in rows. The genes were clustered based on expression similarity; red indicates genes with considerable expression levels, blue denotes genes with low expression levels, and the colour range from red to blue represents large to small values.

The differential expression patterns of the different maturation stages were observed in the heatmaps in Fig. 8 and summarised in Table 8. The *M. rosenbergii* transcriptome annotation results obtained in the current study were mined to identify the genes associated with reproduction and ovary development such as Vg, insulin receptor (IR), oestrogen sulfotransferase (ESULT) and cyclin B (Table 8). Furthermore, there were also first time identified gene found in ovary of *M. rosenbergii*, such as the small ubiquitin-like modifier (SUMO)-activating enzyme subunit 1, E3 ubiquitin-protein ligase RNF25 and neuroparsin. The number of significant differentially regulated genes procured from four ovarian maturity stages of *M. rosenbergii* is indicated in Table 9. Comparison between stages 3 and 1 recorded the most total DEG, 2,114. Other comparisons yielded 1,226 (between stages 2 and 1), 1,429 (between stages 3 and 2), 1,930 (between stages 4 and 1), 1,147 (between stages 4 and 2) and 1,899 (between stages 4 and 3) DEG (Table 9).

**Figure 9:**
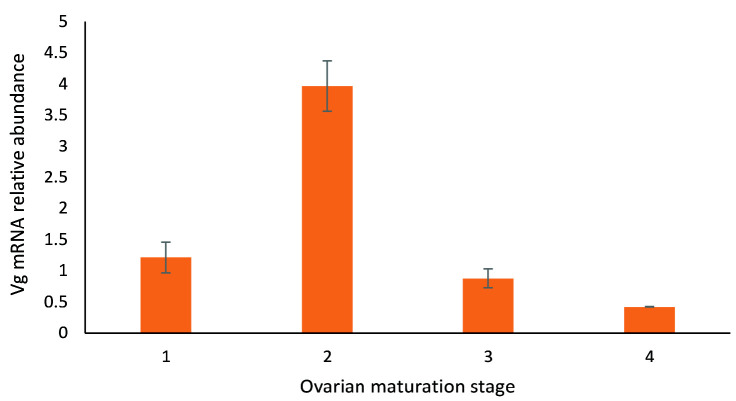
Relative abundance of Vg mRNA transcript in the ovary of *M. rosenbergii* at different ovarian maturation stages. *Note*: 18S rRNA was utilised as the reference gene, and the different alphabet subscripts denote significant differences at *p* < 0.05 according to the pairwise comparison test.

A vital gene associated with reproduction and ovary development maturation is Vg. Based on the heatmap in Fig. 8, Vg expression level was low at stage 1 before increasing during the second stage and decreasing again at the subsequent stages. The Vg levels in the samples evaluated in this study were also validated with qPCR and gene-specific primer (Fig. 9). The result revealed similar upregulation at stages 1 and 2 and downregulation at stages 3 and 4.

## DISCUSSION

The current study identified and analysed various genes and gene expressions in developing ovaries of *M. rosenbergii* samples. Four cDNA libraries were also prepared, each representing the pooled RNA extracted from five prawns of similar maturity phases. The procedure was identical to the report by Waiho *et al*. (2017). The pooled RNA was procured to represent each developmental stage. Pooling minimises biological variation effects (Zhang & Gant 2005) and highlights the substantive gene expressions expressed during each stage (Kendziorski *et al*. 2005). The samples obtained were also employed during differential expression analysis to compare gene expression among stages rather than assessing inter-individual variation within specific stages. The results indicated that varying ovarian maturity phases expressed different particular genes, even when comparing samples from similar tissue types.

A total of 78,103,240, 80,542,030, 89,107,326 and 84,330,036 clean reads were recorded from stages 1, 2, 3 and 4 samples, respectively (Table 3). The clean reads retrieved in the present study were higher than those acquired by Suwansa-Ard *et al*. (2015), at 51,563,078. In another study conducting transcriptome assembly on *M. rosenbergii* ovarian tissues, 6,757,195 clean reads were reported (Jung *et al*. 2016). The assembly programme applied in the current study produced a substantial number of long sequences. For instance, 19,243 unigenes were longer than 1,000 bp, which accounted for 19.63% of the total unigenes, while 18,957 unigenes (19.34 %) were longer than 2,000 bp. The N50 length of the samples was 2,326, which was higher than the value Waiho *et al*. (2019) acquired at 1,225 bp (Table 4). High-quality long sequences enable more information retrieval from the genes.

Table 5 summarises the percentage ratio of successfully annotated genes from the *M. rosenbergii* transcriptome data set produced in this study. Meng *et al*. (2015) suggested that annotated unigenes fundamentally contribute to the *M. rosenbergii* sequence database and set the basis for future investigations on specific molecular processes and functions of the species. In the present study, a significant unigene proportion (61.1%) did yield a BLASTX hit. The limited number of well-annotated protein-coding genes and well-characterised genomes of *M. rosenbergii* or other crustaceans in the public database might explain the observation (Mohd-Shamsudin *et al*. 2013). The non-annotated unigenes in this study were attributable to the 31.90% short-length sequences of 200 bp to 500 bp.

Studies have indicated that most transcriptome databases in decapod crustaceans recorded higher non-annotated gene percentages. For example, Mohd-Shamsudin *et al*. (2013) reported 75% non-annotated gene in *M. rosenbergii*, Meng *et al*. (2015) recorded 77.55% in swimming crab, *Portunus trituberculatus*, Jiang *et al*. (2019) documented 70.7% in *M. rosenbergii* eyestalks, Suwansa-Ard *et al*. (2015) noted 63.7% from eyestalks, central nervous system, and ovaries of *M. rosenbergii*, and Waiho *et al*. (2019) recorded 75% from testes of orange mud crabs, *Scylla olivacea*. The considerable unannotated sequence percentages implied that potentially useful genetic information might be available was missed and remained unexploited. Transcriptomic data might still hold numerous critical genes and valuable genetic information that could be mined (Waiho *et al*. 2019).

The GO terms-based gene distribution obtained in the current study was consistent with previous reports (Jung *et al*. 2011; Zeng *et al*. 2013; Suwansa-Ard *et al*. 2015), indicating that gene encoding the functions are easily annotatable from databases, typically highly conserved throughout evolution, and necessary for multicellular organism survival. Furthermore, systematic unigene classification based on GO, KOG and KEGG biological pathways aimed to identify correlated genes for specific physiological processes and allow comparisons and descriptions of functional features with common terminologies have facilitated biologically meaningful information extractions from high throughput functional genomics data (Mohd-Shamsudin *et al*. 2013). Consequently, gene sequences related to reproduction and ovarian development were identified in *M. rosenbergii* transcriptome data as shown in Table 10.

The ovaries of *M. rosenbergii* develop in four stages. The primary objective of this study was to observe the DEG in each ovarian maturation phase. The data could contribute towards the database for mining novel genes involved in *M. rosenbergii* ovary development, maturation and reproduction. The results revealed that contrasting stages 3 and 1 exhibited the highest total DEG than other stage comparisons (Table 9). The observations might be due to differences in oocyte development at the cellular level between ovaries in stages 1 and 3. During each reproductive cycle, female crustacean gonads undergo a sequence of morphological transformations (Meeratana & Sobhon 2007). The alterations result in various oocyte numbers and classes undergoing numerous cellular differentiation steps (Meeratana & Sobhon 2007). In *M. rosenbergii*, oogenesis and vitellogenesis are the primary events during ovarian maturation. Consequently, each maturation phase would have different genes expressed.

Dakshinamurti (2005) and Jiang *et al*. (2009) hypothesised that different genes are expressed in each spermatogenesis phase and produce proteins with restricted expression patterns. Table 8 lists the selected DEG documented in each maturation stage of the *M. rosenbergii* ovary expression profile. Some genes have already been identified in previous studies, including Vg (Ara & Damrongphol 2014), IR (Sharabi *et al*. 2016), ESULT (Thongbuakaew *et al*. 2016), and cyclin B (Feng *et al*. 2020). The heatmap procured in the present study demonstrated that the expression of cyclin B was low in the first stage of ovarian maturation before rising in the subsequent stages. Cyclin B is critical due to its role with Cdc2 kinase in forming an M-phase promoting factor (MPF), which is central in the meiotic maturation of oocytes. The result supported the findings reported by Feng *et al*. (2020), where cyclin B was expressed in *M. rosenbergii* ovaries during late vitellogenesis and germinal vesicle breakdown (GVBD) stages.

The IR was another differentially expressed gene identified among the ovarian expression profiles assessed in the current study. Although the gene expression was low in the early stage of ovarian maturation, its levels in stages 2 and 3 increased before decreasing in the final stage. IR is a transmembrane receptor belonging to the receptor tyrosine kinases subfamily. In crustaceans, IR functions in insulin signalling pathways, where insulin-like peptides are involved in the principal regulatory sexual differentiation processes and maintenance of sexual characteristics (Ventura *et al*. 2011). The ESULT gene encodes steroidogenic enzymes involved in the steroidogenic pathway. Thongbuakaew *et al*. (2016) identified ESULT in the ovaries of *M. rosenbergii*, exhibiting steroid metabolism roles critical in oogenesis and ovarian development.

The study also found that ESULT plays a crucial component in the oestrogen transport pathway in crustaceans. ESULT adds a sulphate group to oestradiol, rendering it soluble in the haemolymph and enabling its circulation throughout the body (Goodsell 2006). In the present study, ESULT expression was the most significant in stage 2 during ovarian maturation. The Vg is a crucial gene for reproduction and ovary development. The gene was identified in the heatmap obtained in this study. According to Jia *et al*. (2003), total length cDNAs encoding Vg have been identified in approximately 20 crustacean species, such as *M. rosenbergii* (Jayasankar *et al*. 2002), *Metapenaeus ensis* (Kung *et al*. 2004), *Penaeus vannamei* (Parnes *et al*. 2004), *Fenneropenaeus merguiensis* (Auttarat *et al*. 2006), *Penaeus monodon* (Tiu *et al*. 2006), and *Fenneropenaeus chinensis* (Xie *et al*. 2009). The findings suggest that the genes established in the transcriptome data procured in this study were conserved and annotatable from databases.

Based on transcriptome analysis and qPCR performed in the current study, the expression level of Vg was low at stage 1 before increasing at stage 2 and decreasing again at stages 3 and 4. Ovarian development is characterised by accumulating a vital yolk protein (vitellin) and cortical rod formations in oocytes. The Vg, or yolk protein, is the precursor for vitellin, which is present in oviparous animals of almost all species, from nematodes to vertebrates. The Vg supplies proteins, carbohydrates, and lipids to the developing oocytes as resources for the maturing embryo (Jia *et al*. 2013) and combines with metallic ions, including zinc (Zn^2+^), iron (Fe^3+^), copper (Cu^2+^), magnesium (Mg^2^), calcium (Ca^2+^) and carries them into oocytes (Ghosh & Thomas 1995; Montorzi *et al*. 1995). The current study observed previously unreported reproduction and ovarian maturation-related genes in *M. rosenbergii*, for instance, the putative ubiquitin-like modifier (SUMO)-activating enzyme subunit 1.

The SUMO proteins are translated from gene-encoded peptides that conjugate to target proteins. The gene is responsible for controlling various fundamental cellular processes, including cell cycle progression, intracellular trafficking, transcription, DNA repair, embryonic development and organelle biogenesis (Weissman *et al*. 2001; Schmidt & Müller 2003; Hecker *et al*. 2006; Artus *et al*. 2006). The SUMO-1 identified in the present study was downregulated in the early stages of vitellogenesis before being upregulated in stages 2 and 3. Nevertheless, SUMO-1 was downregulated in the final vitellogenesis phase (Table 8). The results were similar to that of *M. japonicas*, in which the expression pattern was highest during the third ovarian maturation stage.

**Figure 10:**
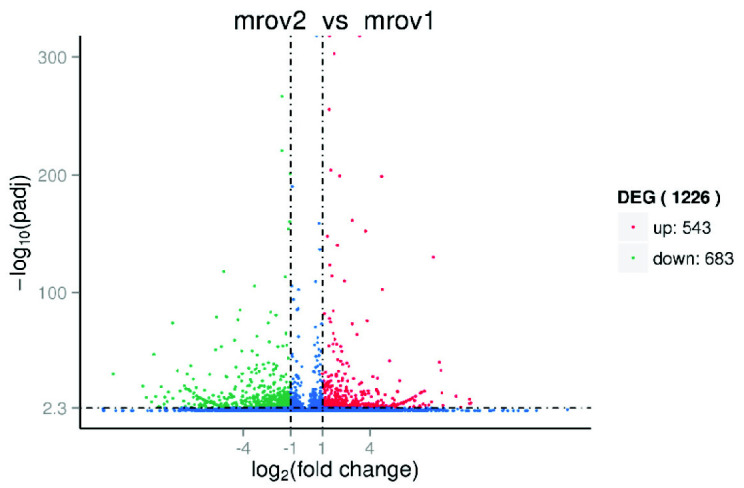

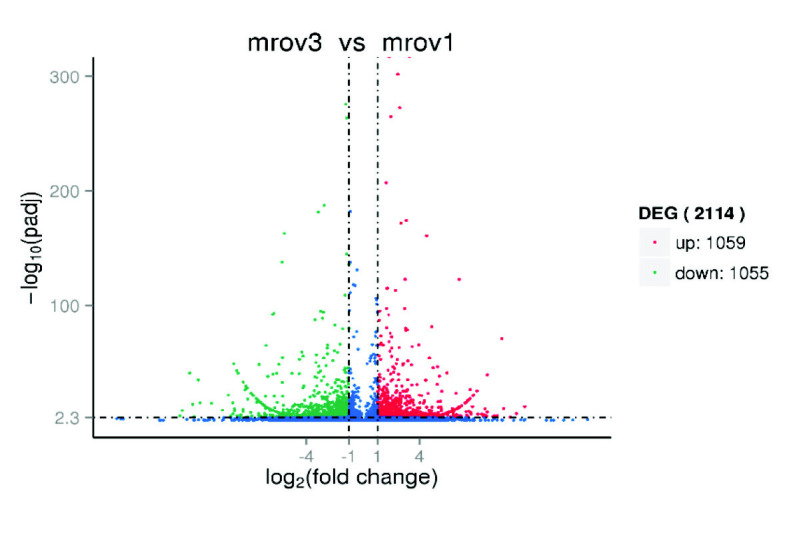

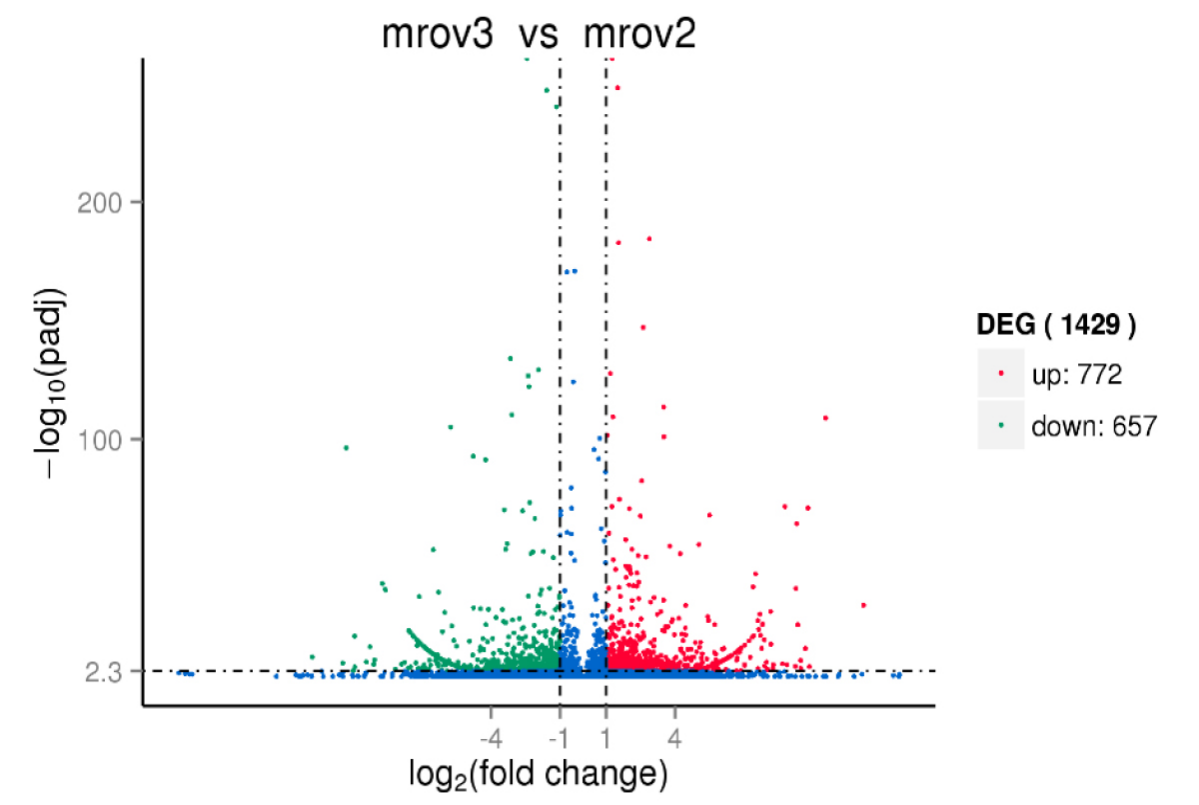

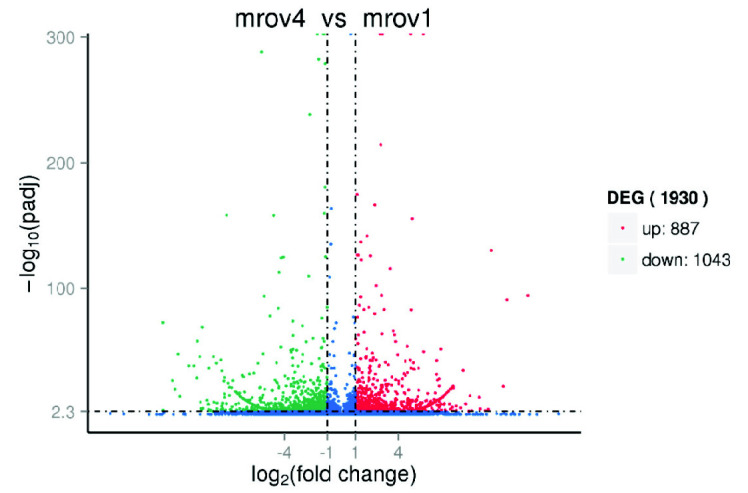
Differential expression analysis in two different stages: (a) expression level of unigenes in Stage 2 versus Stage 1; (b) expression level of unigenes in Stage 3 versus Stage 1; (c) expression level of unigenes in Stage 3 versus 2; (d) expression level of unigenes in Stage 4 versus Stage 1; (e) expression level of unigenes in Stage 4 versus Stage 2; and (f) expression level of unigenes in Stage 4 versus Stage 3. *Notes*: mrov1 = Stage 1 ovary; mrov2 = Stage 2 ovary; mrov3 = Stage 3 ovary; and mrov4 = Stage 4 ovary. The x and y-axis are the log_10_ of the normalised expression level (RPKM) of unigene in the indicated tissue. Red and green points indicate genes with false discovery rate (FDR)-corrected *p*-value < 0.05. Red points indicate up-regulated unigenes and green points indicate down-regulated unigenes in the tissues in which its expression level is represented by the y-axis. Blue points indicate insignificant differentially expressed unigenes.

The findings suggested that specific proteins required for the cell cycle are synthesised, and at stage 3, the proteins are degraded by the E2r-dependent ubiquitin pathway to continue the cell cycle. This study identified E3 ubiquitin-protein ligase RNF25 (Table 8), downregulated at stages 1 and 2 and upregulated at the subsequent stages (Fig. 10). SUMO-specific proteases, including the E3 ubiquitin-protein ligase RNF25, process SUMO proteins to activate and transfer them to the SUMO-conjugating enzyme (Ubc9) (Hashiyama *et al*. 2009). Desterro *et al*. (1997) also reported that Ubc9 mediate SUMOylation by directly binding SUMO to all proteins. The present study is the first to discover SUMO-activating enzyme subunit 1 and E3 ubiquitin-protein ligase RNF25 in *M. rosenbergii* ovaries. Various neurohormones and neurotransmitters control gonad maturation and reproduction. The eyestalk ganglia in crustaceans contain the X-organ or sinus gland complex, the primary source of neuropeptides that inhibit moulting and reproduction (Subramoniam 2000). Numerous genes participate in hormone synthesis and metabolism. Consequently, hormone and biogenic amine receptor-encoding genes are critical for gonadal development and maturation. In the present study, the neuroparsin gene exhibited differential gene expression, upregulated in stages 1 to 3 and downregulated in stage 4 (Table 8). Neuroparsin is a multifunctional neurohormone group that is anti-gonadotropic and primarily found in insects. The hormones regulate haemolymph lipid and trehalose levels and reproduction development.

Yang *et al*. (2015) discovered a crustacean neuroparsin in most vital organs of sand shrimp, *M. ensis*, including the hepatopancreas, nerve cord, brain, heart, ovary and muscle. In the present study, the differential expression pattern of the neuroparsin gene in different stages of ovarian maturation of *M. rosenbergii* indicated that neuroparsin changes in response to GSI alterations (Yang *et al*. 2015). This study also provided evidence of neuroparsin involvement in ovary development. Further analysis of the gene structure, gene family, expression profile during ovarian development, and potential role in reproduction or vitellogenesis could be essential in advancing knowledge of the neuropeptide group, suggesting possible hormone manipulation to improve aquaculture production.

## CONCLUSION

The present study performed the first transcriptome analysis on the different ovarian maturation stages of *M. rosenbergii*. This study successfully yielded 332,082,632 high-quality reads. Furthermore, analysing the prawn transcriptomic data through DEG led to conserved gene identifications, such as cyclin B, IR, ESULT and Vg, vital in *M. rosenbergii* ovarian maturation. Several ovarian-maturation genes were also identified for the first time in the present study, including SUMO-activating enzyme subunit 1, E3 ubiquitin-protein ligase RNF25, and neuroparsin. The genes play critical roles in ovarian maturation. The data obtained in the present study considerably contributes to the knowledge of gene expression and genome structure in *M. rosenbergii* ovaries throughout its developmental stage.

## Figures and Tables

**Table 1:** The RNA-seq adapter sequences employed in the present study.

RNA 5′ adapter	5′AATGATACGGCGACCACCGAGATCTACACTCTTTCCCTACACGAGCCTCTTCCGATCT
RNA 3′ adapter	5′GATCGGAAGAGCACACGTCTGAACTCCAGTCACATCACGATCTCGTATGCCGTCTTCTGCTTG

**Table 2:** The primers employed in the RT-qPCR.

Gene	Primers	Product length (bp)	Reference
Vg	Forward: 5′- CCGGTCACGTGGCGAAGG -3′ Reverse: 5′-ATGCGGACAATCAGAGAAAACA -3′	105	Jayasankar *et al*. (2002)
18 S rRNA	Forward: 5′- TAGCAATTCGCCGTCGTTATTC -3′ Reverse: 5′- CTACCCCCGGAACTCAAAGACT -3′	111	Lafontaine *et al*. (2016)

**Table 3:** The assembly statistics summary.

Sample	Raw read	Clean read	Clean bases	Error (%)	Q20 (%)	Q30 (%)	GC content (%)
Stage 1	81,779,314	78,103,240	11.7G	0.01	97.14	93.14	40.21
Stage 2	84,167,070	80,542,030	12.1G	0.01	97.15	93.12	41.29
Stage 3	91,424,878	89,107,326	13.4G	0.02	96.85	92.35	42.69
Stage 4	87,808,588	84,330,036	12.6G	0.01	97.19	93.18	41.97

**Table 4:** Overview of the length distribution of the unigenes.

Length distribution	Unigenes
Min length	201
Mean length	1,338
Median length	745
Max length	24,939
N50	2,326
N90	548
Total nucleotides	131,093,546

**Table 5:** The number of unigenes successfully annotated genes in *M. rosenbergii* transcriptome data sets.

Annotation database	Number of unigene	Percentage (%)
NR	27,680	28.24
NT	7,448	7.59
KO	13,026	13.29
Swiss-Prot	22,606	23.06
PFAM	29,907	30.51
GO	30,025	30.63
KOG	14,368	14.65
All databases	3,724	3.79
Annotated in at least one database	37,338	38.09

Total unigene	98,013	100.00

**Table 6:** The top five hit species of unigenes in *M. rosenbergii* transcriptome against the NR database.

Scientific name (common name)	Taxanomy			Matched assembled transcript (n)

	Phylum	Subphylum	Class	
*Z. nevadensis* (Nevada termite)	Arthropoda	Hexapoda	Insecta	3,587
*D. pulex* (water fleas)	Arthropoda	Crustacea	Branchiopoda	1,776
*T. castaneum* (red flour beetle)	Arthropoda	Hexapoda	Insecta	923
*S. mimosarum* (spider)	Arthropoda	Chelicerata	Arachnida	895
*B. floridae* (Florida lancelet)	Chordata	Cephalochordata	Leptocardii	866

**Table 7:** The top 20 annotations of *M. rosenbergii* transcriptome with the highest hit score.

Description	Accession ID	Scientific name (common name)	Alignment length (amino acids)	E-value	Hit score	Similarity (%)
Dynein heavy chain, cytoplasmic	KDR21358.1	*Z. nevadensis* (Nevada termite)	4,664	0	18,707	76.92
Cj-cadherin	BAD91056.1	*Caridina japonica*	2,964	0	14,389	91.60
Microtubule-actin cross-linking factor 1 isoform X2	XP_012253403.1	*Athalia rosae*	5,785	0	13,465	47.12
Microtubule-actin cross-linking factor 1 isoform X11	XP_012253413.1	*A. rosae*	5,757	0	13,325	46.93
Microtubule-actin cross-linking factor 1 isoform X26	XP_012253429.1	*A. rosae*	5,699	0	13,313	47.15
Microtubule-actin cross-linking factor 1 isoform X12	XP_012271167.1	*Orussus abietinus*	6,012	0	13,191	45.59
Microtubule-actin cross-linking factor 1 isoform X17	XP_012271173.1	*O. abietinus*	5,839	0	13,174	46.28
Microtubule-actin cross-linking factor 1 isoform X14	XP_012271163.1	*O. abietinus*	5,807	0	13,052	46.12
Pre-mRNA-processing-splicing factor 8	XP_012255140.1	*A. rosae*	2,371	0	11,179	87.22
Target of rapamycin	AHX84170.1	*Fenneropenaeus chinensis*	2,473	0	10,921	86.66
Spectrin beta chain, non-erythrocytic 1 isoform X3	XP_012268006.1	*A. rosae*	3,892	0	10,882	55.04
Spectrin alpha chain	KDR23504.1	*Z. nevadensis*	2,430	0	7,695	81.40
Neurofibromin	XP_008200683.1	*T. castaneum*	2,509	0	9,716	76.52
Vg receptor	ADK55596.1	*M. rosenbergii*	1,677	0	9,107	97.79
Spectrin beta chain	XP_008470128.1	*Diaphorina citri*	2,303	0	8,542	72.08
Fat-like cadherin-related tumour suppressor homolog, partial	XP_011135487.1	*Harpegnathos saltator*	4,421	0	8,329	41.46

**Table 8:** Selected DEG between ovarian maturation stages in the *M. rosenbergii* samples evaluated.

Cluster on heatmap	Gene name	Log2FC	*p*-value	Regulation	Accession ID of hit
Stage 2 vs 1					

Cluster-13227.41540	RNA-directed DNA polymerase (reverse transcriptase) domain containing protein	8.3563	1.54E-44	+	CDJ92648.1
Cluster-13227.29274	Protein transport protein Sec16A	9.7366	3.03E-05	+	KFM6013
Cluster-13227.27176	SUMO-activating enzyme subunit 1	5.5475	4.42E-07	+	AGC75066.1
Cluster-13227.27547	Serine proteinase inhibitor	7.9913	4.85E-134	+	BAI50776.1
Cluster-13227.23038	IR	2.319	1.14E-06	+	CDI30232.1
Cluster-13227.47174	Ecdysteroid-regulated 16 kDa protein	2.2662	9.22E-43	+	XP_975622.1
Cluster-13227.36029	Cyclin B	2.1483	2.27E-45	+	ACI46952.1
Cluster-13227.13468	ESULT	2.5479	3.46E-05	+	AJC52502.1
Cluster-13227.25663	Tensin	8.6171	5.05E-07	+	KDR23674.1
Cluster-13227.33929	Cyclooxygenase [*P. monodon*]	1.8551	1.89E-13		AHA44500.1
Cluster-13227.24564	Trichohyalin isoform X5	−9.2755	2.86E-05	−	XP_009295459.1
Cluster-13227.30080	MICOS complex subunit Mic10	−7.7313	2.01E-24	−	XP_003729965.1
Cluster-13227.26920	DNA-directed RNA polymerase II subunit RPB7	−7.6287	9.73E-22	−	KFM82434.1
Cluster-13227.21798	Protein DAPPUDRAFT_191117	−7.2974	1.72E-05	−	EFX89037.1
Cluster-13227.35628	ATP-dependent RNA helicase WM6	−7.1591	4.17E-30	−	KDR23694.1
Cluster-13227.23329	Zinc metalloproteinase nas-4-like	−7.0983	7.71E-08	−	XP_011692983.1

Stage 3 vs 1					

Cluster-13227.31753	Vg receptor	2.6044	4.46E-277	+	ADK55596.1
Cluster-13227.29274	Protein transport protein Sec16A	10.786	4.40E-08	+	KFM60130.1
Cluster-13227.52182	Pre-mRNA 3′ end processing protein WDR33	10.348	9.51E-05	+	XP_007956037.1
Cluster-13227.6492	Preproinsulin 2	7.3522	1.06E-19	+	ABB89749.1
Cluster-13227.23038	IR	2.0209	9.23E-05	+	CDI30232.1
Cluster-13227.28010	E3 ubiquitin-protein ligase RNF25	5.7518	7.44E-08	+	KDR23110.1
Cluster-13227.30023	PDZ and LIM domain protein Zasp	6.2576	1.64E-10	+	XP_012546054.1
Cluster-13227.30855	Selenoprotein K	−12.711	4.70E-11	−	XP_785269.3
Cluster-13227.39872	Soluble calcium-activated nucleotidase 1	−11.862	2.34E-05	−	KFM65553.1
Cluster-13227.39431	Serine proteinase inhibitor	−6.5141	2.49E-12	−	BAI50776.1
Cluster-13227.41063	Dynactin subunit 4	−4.8838	7.39E-05	−	KDR09402.1
Cluster-15558.0	Transposon-derived buster3 transposase-like protein-like	−5.1686	2.24E-05	−	XP_003737393.1

Stage 3 vs Stage 2					

Cluster-13227.32963	Serine proteinase inhibitor	10.539	3.96E-113	+	BAI50776.1
Cluster-13227.56974	CUB-serine protease	9.3236	4.12E-25	+	AAK48894.1
Cluster-13227.34016	Ovochymase-2 precursor	9.2482	1.20E-40	+	NP_001081896.1
Cluster-13227.24311	GST-N-metaxin-like protein	8.6603	5.99E-08	+	EFX78317.1
Cluster-13227.32770	Vg receptor	1.2587	1.31E-28	+	ADK55596.1
Cluster-13227.27176	SUMO-activating enzyme subunit 1	1.8859	1.33E-09	+	AGC75066.1
Cluster-13227.32821	Cathepsin A	5.4918	9.74E-07	+	ADO65982.1
Cluster-13227.35787	Microtubule-actin cross-linking factor 1 isoform X2	5.4338	1.62E-06	+	XP_012253403.1
Cluster-13227.27547	Serine proteinase inhibitor	−10.287	2.04E-100	−	BAI50776.1
Cluster-13227.37715	Probable small nuclear ribonucleoprotein Sm D2	−9.0968	1.17E-07	−	XP_005099877.1
Cluster-13227.38643	Protein hu-li tai shao	−6.0517	1.72E-09	−	KDR18780.1
Cluster-13227.40864	Neuroparsin	−2.2919	1.77E-05	−	AHG98659.1
Cluster-13227.55705	Carbohydrate sulfotransferase 11	−6.6925	2.62E-05	−	KDR09457.1

Stage 4 vs Stage 1					

Cluster-13227.29274	Protein transport protein Sec16A, partial	10.306	1.83E-06	+	KFM60130.1
Cluster-13227.14589	protein DAPPUDRAFT_205678	7.8424	4.60E-24	+	EFX89253.1
Cluster-13227.31753	Vg receptor	1.5835	5.53E-69	+	ADK55596.1
Cluster-13227.24612	Vg 2	1.1314	7.25E-08	+	AHD26978.1
Cluster-13227.27679	Ubiquitin-activating enzyme E1	1.2197	2.06E-09	+	AFK65746.1
Cluster-13227.36029	Cyclin B	1.7927	8.91E-26	+	ACI46952.1
Cluster-13227.38572	Cyclin k	−12.491	1.47E-05	−	XP_002434453.1
Cluster-13227.31315	Antioxidant enzyme	−9.2984	3.81E-40	−	XP_001865898.1
Cluster-13227.31929	Serine proteinase-like protein	−3.7178	2.53E-14	−	AFI61881.1
Cluster-13227.32963	Serine proteinase inhibitor	−7.6639	1.12E-08	−	BAI50776.1
Cluster-13227.32945	Will die slowly	−1.4091	6.72E-06	−	AGO01382.1

Stage 4 vs Stage 2					

Cluster-13227.43662	RNA-directed DNA polymerase (reverse transcriptase) domain containing protein	3.8795	6.29E-35	+	CDJ92648.1
Cluster-13227.43859	Phospholipase D	7.0788	1.16E-16	+	XP_011132527.1
Cluster-13227.35787	Microtubule-actin cross-linking factor 1 isoform X2	5.9752	8.07E-09	+	XP_012253403.1
Cluster-13227.34020	Transforming growth factor-beta-induced protein ig-h3	1.8192	1.22E-05	+	KDR10948.1
Cluster-13227.31856	Kinesin heavy chain	1.2352	7.67E-06	+	EFA10675.1
Cluster-13227.38572	Cyclin k	−12.116	7.69E-05	−	XP_002434453.1
Cluster-13227.38077	SH3 domain-containing kinase-binding protein 1	−9.2988	5.75E-05	−	ELK12901.1
Cluster-13227.38122	Protein DAPPUDRAFT_300659	−9.1719	1.64E-55	−	EFX69840.1
Cluster-13227.31315	Antioxidant enzyme	−9.1411	3.61E-37	−	XP_001865898.1
Cluster-13227.31711	E3 ubiquitin-protein ligase RNF25	−5.0626	1.91E-05	−	KDR23110.1

Stage 4 vs Stage 3					

Cluster-13227.30855	Selenoprotein K	11.434	2.36E-06	+	XP_785269.3
Cluster-13227.38572	Cyclin k	−12.679	7.41E-06	−	XP_002434453.1
Cluster-13227.32963	Serine proteinase inhibitor	−12.191	2.22E-85	−	BAI50776.1
Cluster-13227.56974	CUB-serine protease	−10.975	6.79E-20	−	AAK48894.1
Cluster-13227.39355	Serine proteinase inhibitor	−10.092	7.25E-90	−	BAI50776.1
Cluster-13227.47615	Egg-derived tyrosine phosphatase	−1.1138	3.04E-07	−	ETN58617.1

**Table 9:** Differential expression analysis of *M. rosenbergii* at gene level.

Significant DEG				

Cond 1	Cond 2	Total (differentially regulated unigenes)	Up-regulation (Cond. 2 > Cond. 1)	Down-regulation (Cond. 2 < Cond. 1)
Stage 2	Stage 1	1,226	543	683
Stage 3	Stage 1	2,114	1,059	1,055
Stage 3	Stage 2	1,429	772	657
Stage 4	Stage 1	1,930	887	1,043
Stage 4	Stage 2	1,147	569	578
Stage 4	Stage 3	1,899	830	1,069

*Note*: Cond = condition.

**Table 10:** The selected reproduction regulators and ovary development-related genes.

Identity	Accession ID	Hit organism	Similarity (%)	E-value	Example of query ID
Cyclin B	ADP95148.1	*M. rosenbergii*	94.00	3.8e-205	Cluster-13227.7667
Cathepsin L	AGN52717.1	*M. rosenbergii*	93.86	1.10e-189	Cluster-13227.31421
Insulin-like receptor	AKF17681.1	*M. rosenbergii*	93.23		Cluster-13227.36037
Gonadotropin-releasing hormone receptor	AHB33640.1	*Macrobrachium nipponense*	89.75	1.10e-187	Cluster-13227.22181
Mitogen-activated protein kinase kinase	AHA93093.1	*Scylla paramamosain*	89.41	1.60e-209	Cluster-13227.34441
Vg	AJP60219.1	*M. nipponense*	89.37	5.10e-102	Cluster-5462.0
Heat shock protein 90	CDF32000.1	*M. rosenbergii*	88.76	1.70e-79	Cluster-4043.0
Eka-protein kinase A protein	CFW94247.1	*Euperipatoides kanangrensis*	87.43	7.10e-18	Cluster-13227.14623
Oestrogen-related receptor	ADB43256.1	*S. paramamosain*	87.20	3.8e-226	Cluster-13227.6195
Vasa-like protein	ADB28894.1	*M. nipponense*	85.69	1.30e-292	Cluster-13227.30269
Vg receptor	ADK55596.1	*M. rosenbergii*	84.51	1.4e-99	Cluster-13227.32770
Cyclooxygenase	AHA44500.1	*Penaeus monodon*	82.35	1.70e-70	Cluster-26293.0

## References

[b1-tlsr_35-3-77] Ara F, Damrongphol P (2014). Vitellogenin gene expression at different ovarian stages in the giant freshwater prawn, *Macrobrachium rosenbergii*, and stimulation by 4-nonylphenol. Aquaculture Research.

[b2-tlsr_35-3-77] Arockiaraj J, Easwvaran S, Vanaraja P, Singh A, Othman RY, Bhassu S (2012). Molecular cloning, characterization and gene expression of an antioxidant enzyme catalase (*M*rCat) from *Macrobrachium rosenbergii*. Fish and Shellfish Immunology.

[b3-tlsr_35-3-77] Arockiaraj J, Vanaraja P, Easwvaran S, Singh A, Alinejaid T, Othman RY, Bhassu S (2011). Gene profiling and characterization of arginine kinase-1 (MrAK-1) from freshwater giant prawn (*Macrobrachium rosenbergii*). Fish and Shellfish Immunology.

[b4-tlsr_35-3-77] Arockiaraj M, Manuel P, Rajasingh I, Rajan B (2011). Wirelength of 1-fault hamiltonian graphs into wheels and fans. Information Processing Letters.

[b5-tlsr_35-3-77] Artus J, Babinet C, Cohen-Tannoudji M (2006). The cell cycle of early mammalian embryos lessons from genetic mouse models. Cell Cycle.

[b6-tlsr_35-3-77] Auttarat J, Phiriyangkul P, Utarabhand P (2006). Characterization of vitellin from the ovaries of the banana shrimp *Litopenaeus merguiensis*. Comparative Biochemistry and Physiology Part B: Biochemistry and Molecular Biology.

[b7-tlsr_35-3-77] Baliarsingh S, Chung JM, Sahoo S, Sarkar A, Mohanty J, Han YS, Lee YS, Patnaik BB (2021). Transcriptome analysis of *Macrobrachium rosenbergii* hepatopancreas in response to *Vibrio harveyi* infection. Aquaculture Research.

[b8-tlsr_35-3-77] Dakshinamurti K (2005). Biotin: A regulator of gene expression. The Journal of Nutritional Biochemistry.

[b9-tlsr_35-3-77] De Grave S, Cai Y, Anker A (2008). Global diversity of shrimps (Crustacea: Decapoda: Caridea) in freshwater. Hydrobiologia.

[b10-tlsr_35-3-77] Desterro JMP, Thomson J, Hay RT (1997). Ubc9 conjugates SUMO but not ubiquitin. FEBS Letter.

[b11-tlsr_35-3-77] Ding Z, Jin M, Ren Q (2018). Transcriptome analysis of *Macrobrachium rosenbergii* intestines under the white spot syndrome virus and poly (I:C) challenges. PLoS One.

[b12-tlsr_35-3-77] Feng H, Dong YT, Liu X, Qiu GF (2020). Cyclin B protein undergoes increased expression and nuclear relocation during oocyte meiotic maturation of the freshwater prawn *Macrobrachium rosenbergii* and the Chinese mitten crab *Eriocheir sinensis*. Gene.

[b13-tlsr_35-3-77] Ghosh P, Thomas P (1995). Binding of metals to red drum vitellogenin and incorporation into oocytes. Marine Environmental Research.

[b14-tlsr_35-3-77] Goodsell DS (2006). The molecular perspective: Cisplatin. Stem Cells.

[b15-tlsr_35-3-77] Götz S, García-Gómez JM, Terol J, Williams TD, Nagaraj SH, Nueda MJ, Robles M, Talón M, Dopazo J, Conesa A (2008). High-throughput functional annotation and data mining with the Blast2GO suite. Nucleic Acids Research.

[b16-tlsr_35-3-77] Grabherr MG, Haas BJ, Yassour M, Levin JZ, Thompson DA, Amit I, Adiconis A (2011). Full-length transcriptome assembly from RNA-Seq data without a reference genome. Nature Biotechnology.

[b17-tlsr_35-3-77] Hashiyama K, Shigenobu S, Kobayashi S (2009). Expression of genes involved in sumoylation in the Drosophila germline. Gene Expression Patterns.

[b18-tlsr_35-3-77] Hecker CM, Rabiller M, Haglund K, Bayer P, Dikic I (2006). Specification of SUMO1-and SUMO2-interacting Motifs. Journal of Biological Chemistry.

[b19-tlsr_35-3-77] Jayasankar V, Tsutsui N, Jasmani S, Saido-Sakanaka H, Yang WJ, Okuno A, Hien TTT, Aida K, Wilder MN (2002). Dynamics of vitellogenin mRNA expression and changes in hemolymph vitellogenin levels during ovarian maturation in the giant freshwater prawn *Macrobrachium rosenbergii*. Journal of Experimental Zoology.

[b20-tlsr_35-3-77] Jia X, Chen Y, Zou Z, Lin P, Wang Y, Zhang Z (2013). Characterization and expression profile of Vitellogenin gene from *Scylla paramamosain*. Gene.

[b21-tlsr_35-3-77] Jiang H, Yin Y, Zhang X, Hu S, Wang Q (2009). Chasing relationships between nutrition and reproduction: A comparative transcriptome analysis of hepatopancreas and testis from *Eriocheir sinensis*. Comparative Biochemistry and Physiology Part D: Genomics and Proteomics.

[b22-tlsr_35-3-77] Jiang Q, Min Y, Yang H, Wan W, Zhang X (2019). De novo transcriptome analysis of eyestalk reveals ovarian maturation related genes in *Macrobrachium rosenbergii*. Aquaculture.

[b23-tlsr_35-3-77] Jung H, Lyons RE, Dinh H, Hurwood DA, McWilliam S, Mather PB (2011). Transcriptomics of a giant freshwater prawn (*Macrobrachium rosenbergii*): De novo assembly, annotation and marker discovery. PLoS ONE.

[b24-tlsr_35-3-77] Jung H, Yoon BH, Kim WJ, Kim DW, Hurwood DA, Lyons RE, Salin KR, Kim H, Baek I, Chand V, Mather PB (2016). Optimizing hybrid de novo transcriptome assembly and extending genomic resources for giant freshwater prawns (*Macrobrachium rosenbergii*): The identification of genes and markers associated with reproduction. International Journal of Molecular Sciences.

[b25-tlsr_35-3-77] Kendziorski C, Irizarry RA, Chen KS, Haag JD, Gould MN (2005). On the utility of pooling biological samples in microarray experiments. Proceedings of the National Academy of Sciences.

[b26-tlsr_35-3-77] Kung SY, Chan SM, Hui JHL, Tsang WS, Mak A, He JG (2004). Vitellogenesis in the sand shrimp, *Metapenaeus ensis*: The contribution from the hepatopancreas-specific vitellogenin gene (*MeVg2).*. Biology of Reproduction.

[b27-tlsr_35-3-77] Lafontaine A, Hanikenne M, Boulangé-Lecomte C, Forget-Leray J, Thomé JP, Gismondi E (2016). Vitellogenin and vitellogenin receptor gene expression and 20-hydroxyecdysone concentration in *Macrobrachium rosenbergii* exposed to chlordecone. Environmental Science and Pollution Research.

[b28-tlsr_35-3-77] Liu X, Jiang H, Ye B, Qian H, Guo Z, Bai H, Gong J, Feng J, Ma K (2021). Comparative transcriptome analysis of the gills and hepatopancreas from *Macrobrachium rosenbergii* exposed to the heavy metal Cadmium (Cd^2+^). Scientific Reports.

[b29-tlsr_35-3-77] Livak KJ, Schmittgen TD (2001). Analysis of relative gene expression data using real-time quantitative PCR and the 2^−ΔΔCT^ method. Methods.

[b30-tlsr_35-3-77] Martins J, Ribeiro K, Rangel-Figueiredo T, Coimbra J (2007). Reproductive cycle, ovarian development, and vertebrate-type steroids profile in the freshwater prawn *Macrobrachium rosenbergii*. Journal of Crustacean Biology.

[b31-tlsr_35-3-77] Meeratana P, Sobhon P (2007). Classification of differentiating oocytes during ovarian cycle in the giant freshwater prawn, *Macrobrachium rosenbergii* De Man. Aquaculture.

[b32-tlsr_35-3-77] Meng XL, Liu P, Jia FL, Li J, Gao BQ (2015). *De novo* transcriptome analysis of *Portunus trituberculatus* ovary and testis by RNA-Seq: Identification of genes involved in gonadal development. PLoS ONE.

[b33-tlsr_35-3-77] Mohd-Shamsudin MI, Kang Y, Lili Z, Tan TT, Kwong QB, Liu H, Zhang G, Othman RY, Bhassu S (2013). In-depth tanscriptomic analysis on giant freshwater prawns. PLoS ONE.

[b34-tlsr_35-3-77] Montorzi M, Falchuk KH, Vallee BL (1995). Vitellogenin and lipovitellin: Zinc proteins of *Xenopus laevis* oocytes. Biochemistry.

[b35-tlsr_35-3-77] Parnes S, Mills E, Segall C, Raviv S, Davis C, Sagi A (2004). Reproductive readiness of the shrimp *Litopenaeus vannamei* grown in a brackish water system. Aquaculture.

[b36-tlsr_35-3-77] Pasookhush P, Hindmarch C, Sithigorngul P, Longyant S, Bendena WG, Chaivisuthangkura P (2019). Transcriptomic analysis of *Macrobrachium rosenbergii* (giant freshwater prawn) post-larvae in response to *M. rosenbergii* nodavirus (*Mr*NV) infection: De novo assembly and functional annotation. BMC Genomics.

[b37-tlsr_35-3-77] Rao R, Zhu YB, Alinejad T, Tiruvayipati S, Lin Thong KL, Wang J, Bhassu S (2015). RNA-seq analysis of *Macrobrachium rosenbergii* hepatopancreas in response to *Vibrio parahaemolyticus* infection. Gut Pathogens.

[b38-tlsr_35-3-77] Schmidt D, Muller S (2003). PIAS/SUMO: New partners in transcriptional regulation. Cellular and Molecular Life Sciences.

[b39-tlsr_35-3-77] Sharabi O, Manor R, Weil S, Aflalo ED, Lezer Y, Levy T, Aizen J (2016). Identification and characterization of an insulin-like receptor involved in crustacean reproduction. Endocrinology.

[b40-tlsr_35-3-77] Soonklang N, Wanichanon C, Stewart MJ, Stewart P, Meeratana P, Hanna PJ, Sobhon P (2012). Ultrastructure of differentiating oocytes and vitellogenesis in the giant freshwater prawn, *Macrobrachium rosenbergii* (De Man). Microscopy Research and Technique.

[b41-tlsr_35-3-77] Storey JD (2003). The positive false discovery rate: A Bayesian interpretation and the q-value. The Annals of Statistics.

[b42-tlsr_35-3-77] Subramoniam T (2000). Crustacean ecdysteroids in reproduction and embryogenesis. Comparative Biochemistry and Physiology Part C: Pharmacology, Toxicology and Endocrinology.

[b43-tlsr_35-3-77] Suwansa-Ard S, Thongbuakaew T, Wang T, Zhao M, Elizur A, Hanna PJ, Sretarugsa P, Cummins SF, Sobhon P (2015). *In silico* neuropeptidome of female *Macrobrachium rosenbergii* based on transcriptome and peptide mining of eyestalk, central nervous system and ovary. PLoS ONE.

[b44-tlsr_35-3-77] Thongbuakaew T, Siangcham T, Suwansa-Ard S, Elizur A, Cummins SF, Sobhon P, Sretarugsa P (2016). Steroids and genes related to steroid biosynthesis in the female giant freshwater prawn, *Macrobrachium rosenbergii.*. Steroids.

[b45-tlsr_35-3-77] Tiu SHK, Hui JHVL, Mak ASC, He JG, Chan SM (2006). Equal contribution of hepatopancreas and ovary to the production of vitellogenin (PmVg 1) transcripts in the tiger shrimp, *Penaeus monodon*. Aquaculture.

[b46-tlsr_35-3-77] Ventura T, Rosen O, Sagi A (2011). From the discovery of the crustacean androgenic gland to the insulin-like hormone in six decades. General and Comparative Endocrinology.

[b47-tlsr_35-3-77] Waiho K, Fazhan H, Shahreza MS, Moh JHZ, Noorbaiduri S, Wong LL, Sinnasamy S, Ikhwanuddin M (2017). Transcriptome analysis and differential gene expression on the testis of orange mud crab, *Scylla olivacea*, during sexual maturation. PLoS ONE.

[b48-tlsr_35-3-77] Waiho K, Shi X, Fazhan H, Li S, Zhang Y, Zheng H, Liu W, Fang S, Ikhwanuddin M, Ma H (2019). High-density genetic linkage maps provide novel insights into ZW/ZZ sex determination system and growth performance in mud crab (*Scylla paramamosain*). Frontiers in Genetics.

[b49-tlsr_35-3-77] Weissman IL, Anderson DJ, Gage F (2001). Stem and progenitor cells: Origins, phenotypes, lineage commitments, and transdifferentiations. Annual Review of Cell and Developmental Biology.

[b50-tlsr_35-3-77] Xie S, Sun L, Liu F, Dong B (2009). Molecular characterization and mRNA transcript profile of vitellogenin in Chinese shrimp, *Fenneropenaeus chinensis*. Molecular Biology Reports.

[b51-tlsr_35-3-77] Yang BZ, Yang L, Zhang P, Tan YG, Yan L, Chen S (2015). Fish by-catch in shrimp beam trawls in the northern South China Sea. Journal of Applied Ichthyology.

[b52-tlsr_35-3-77] Ying N, Wang Y, Song X, Qin B, Wu Y, Yang L, Fang W (2022). Transcriptome analysis of *Macrobrachium rosenbergii*: Identification of precocious puberty and slow-growing information. Journal of Invertebrate Pathology.

[b53-tlsr_35-3-77] Zeng D, Chen X, Xie D, Zhao Y, Yang C, Li Y, Ma N, Peng M, Yang Q, Liao Z, Wang H, Chen X (2013). Transcriptome analysis of Pacific white shrimp (*Litopenaeus vannamei*) hepatopancreas in response to Taura syndrome Virus (TSV) experimental infection. PLoS ONE.

[b54-tlsr_35-3-77] Zhang SD, Gant TW (2005). Effect of pooling samples on the efficiency of comparative studies using microarrays. Bioinformatics.

